# A study to determine and compare the knowledge, attitude and compliance of Tuberculosis treatment among HIV seropositive and HIV seronegative TB patients

**DOI:** 10.1186/1471-2334-14-S3-P9

**Published:** 2014-05-27

**Authors:** S Gouda, B Peerapur, J Rudramma, A Kaleem, R Sandhya

**Affiliations:** 1 Department of Microbiology, Raichur Institute of Medical Sciences, Raichur, India; 2 Department of Community Medicine, Raichur Institute of Medical Sciences, Raichur, India

## Background

Tuberculosis and HIV are major global health problems. Tuberculosis is the most common opportunistic infection, leading to the mortality among HIV patients. This study aims to determine and compare the knowledge, attitude, and compliance regarding tuberculosis amongst HIV seropositive and HIV seronegative TB patients.

## Methods

This study was conducted at Raichur district hospital. Data regarding socio demographic profile, knowledge, attitude, and compliance was collected from the subjects with the help of pretested semi structured questionnaire. Data was analyzed using Microsoft Excel and SPSS 15.0 and Chi square test was applied.

## Results

A total of 240 subjects aged 19-67 years were enrolled in the study. Out of them, 120 were HIV seropositive, 53% were male, and 68% had primary education. About 55.4% had average knowledge of TB but in depth knowledge about symptoms and the spread was not adequate among HIV seropositive patients. Regarding knowledge on the symptoms and spread of TB, only 35% knew about chest pain and 23% knew about hemoptysis. There were few misconceptions regarding the spread; majority (77%) believed that TB spreads by unprotected sex and 53.5% believed that it occurs through infected blood transfusion. We noticed positive attitude and positive compliance with TB treatment among majority of the respondents, however the compliance was better (92%) among HIV seropositive patients.

## Conclusion

Our study revealed that the knowledge regarding Tuberculosis among HIV seropositive patients is inadequate when compared to HIV seronegative patients. This calls for an awareness program targeting the HIV/AIDS infected individuals through advocacy, communication, and social mobilization.

